# An Adaptive System for Home Monitoring Using a Multiagent Classification of Patterns

**DOI:** 10.1155/2008/136054

**Published:** 2008-04-17

**Authors:** Ali Rammal, Sylvie Trouilhet, Nicolas Singer, Jean-Marie Pécatte

**Affiliations:** ^1^IRIT-Centre Universitaire de information et d' Recherche (CUFR) Jean-François Champollion, site de Castres, Avenue Georges Pompidou, 81100 Castres, France; ^2^IRIT, Université Paul Sabatier (UPS), site de Castres, Avenue Georges Pompidou, 81100 Castres, France

## Abstract

This research takes place in the S(MA)^2^D project which proposes software architecture to monitor elderly people in their own homes. We want to build patterns dynamically from data about activity, movements, and physiological information of the monitored people. To achieve that, we propose a multiagent method of classification: every agent has a simple know-how of classification. Data generated at this local level are communicated and adjusted between agents to obtain a set of patterns. The patterns are used at a personal level, for example to raise an alert, but also to evaluate global risks (epidemic, heat wave). These data are dynamic; the system has to maintain the built patterns and has to create new patterns. So, the system is adaptive and can be spread on a large scale.

## 1. INTRODUCTION

In Europe, many countries will be confronted with aging populations in the coming decades. For example, it is estimated that in 2020, 28% of the French population will be over 60 [1]. A great way to resolve partially this difficulty is to encourage old people to be cared for in their own homes. This strategy presents two main advantages:

- the elderly want to stay at home as long as possible; they keep the privacy they do not want to lose,
- it is less expensive than a place in a collective accommodation.

Our project takes place in this context. It aims to help professional home-care teams in their job by thinking up innovative software technologies, more precisely:

- by increasing the number of old people looked after in their homes with an adaptive and nonintrusive remote assistance,
- by reassuring family circle. The system ensures that the monitored person is secure; so, people around him feel at ease, and
- by contributing towards its democratization. The use of simple elements (e.g., basic sensors) minimizes the initial cost of a monitoring system.

We made a study of systems having the same aim—the following section describes three well-known and relevant European systems in the home-care domain. These systems focus on individuals (they are user-centred): a system surveys only one person; thus, there is a duplication for each individual looked after. None of these systems collects individual monitoring for merging global behaviour patterns. Nevertheless, patterns of monitored people could be used to estimate the status of someone in relation to their community or to integrate new comings.

We propose a multiagent system that is able to generalize, which builds a classification of monitored people. An agent watches over one or more indicators of a group of people. An indicator is data about daily activities, positions, and physiological information. In a first step, the agent applies a local-classification method and obtains an incomplete patterns' partition. Next, the partial partitions are compared with each other in order to build a complete classification.

We conceived an open system: new people or/and new indicators bring in new agents or/and new patterns.

In Section 3, we present the architecture of the system and how it runs.

The system manages a set of patterns of monitored people. This dynamically updated classification has the following three main uses:

- to find certain similarities with the existing tools for evaluating the dependence—dependence grid of the social services, for example,
- to get global statistical data about old people looked after in their own homes, and
- to generate specialized alarms depending on the detected event. Once the classification is set up and people status is known, decisions can be taken to personalize the process of monitoring someone—activated sensors, generated alarms, and danger zone.

These aspects are discussed in the last section.

## 2. A SURVEY OF THREE HOME-MONITORING SYSTEMS

The use of computers to help people stay at home has been the subject of many research projects. Some of them are quite ambitious and regroup many partners. In this section, we describe a selection of three projects designed to assist people in their living environment. We expect to give the reader an overview of the advancement in this area and also the bases our project is laying on.

The selection shows different hardware and software problematics (communication networks, system interoperability, data analysis, emergency handling, and alerts filtering). These problems must be solved to achieve efficient monitoring. We begin by explaining the main objectives of each project. Then, we propose a table that summarizes their most relevant features.

### 2.1. The PROSAFE project

The PROSAFE project [2, 3] attempts to automatically identify the daily activities of the monitored person. The processing of collected data is carried out on doctor's request with an adapted interface.

The final operational objective is to detect any abnormal behaviour such as a fall, a runaway, or an accident. The research objective is to gather characteristic data about the nightly or daily activities of the patient. More precisely, the system can

- describe events that took place during monitoring time—time spent in bed or in the toilets, entering or leaving the bedroom, moving inside the home,
- build a database with all abnormal situations detected, and
- build statistics about past activities.

At the hardware level, the system configuration uses a ground network (a mobile version is also usable). Currently acquisition and data processing are local, and monitoring is both local and distant.

The PROSAFE system is primary used by the medical staff in hospitals. The interface for nurses allows them visualizing the patient state and abnormal situations (alerts and alarms) in the bedroom. As soon as an alarm is raised, a beeper calls a nurse. In the same time, doctors can access a database updated in real time with statistical data about the patient behaviour.

Experiments have been made to gather data about the daily activities of patients in hospitals, especially during the night. Experimental sites have been set up in two hospitals and three more are being installed in elderly people residences.

To conclude, let us say that one of the main features of this project is to be based on real-time analysis of data.

### 2.2. The AILISA project

The AILISA project [4, 5] (Intelligent Apartments for effective longevity) is an experimental platform to evaluate remote care and assistive technologies in gerontology. This ambitious project regroups specialists of smart homes, networks and computing, electronics, and signal processing. More precisely, the project sets up a monitoring platform composed of

- a home equipped with a set of sensors and health devices (presence detectors, wrist arterial pressure sensors, and pulse oximeter),
- a smart shirt developed by the French company TAM with several sensors and electronics embedded in the textile to detect falls,
- a smart assistant robot for ambulation to secure the displacements and assist the person during transfers, and
- a software system to gather and analyze the sensors output.

The project aims to set up an interdisciplinary platform for the evaluation of the technologies at the three following levels: technical, medical, and ethical.

### 2.3. The e-Vital project

The e-Vital project [6] (cost-effective health services for interactive continuous monitoring of vital signs parameters) is a modular and ambulatory telemedicine platform. Its objective is to increase patient's feeling of safety concerning their health. Patients and caregivers feed a central database with some measuring equipment. The developed device allows staff to take measurements and data collected to be sent to the resident doctor. This doctor can remotely diagnose whether there is a problem that needs them to visit or that requires the resident to receive hospital treatment.

By way of a personal digital assistant (PDA), the e-Vital server connects monitoring devices produced by several manufacturers. The server is a multiagent system where each agent focuses on a specific task related to the medical stored data. For example, an alert manager is specialized for the raising of alert messages, a profile manager for access management and a schedule manager for healthcare scheduling.

The e-Vital project is mainly hardware and tries to solve the interoperability problems between non compatible devices. It focuses on communication protocols and on the central database format.

These objectives (care protocol, devices interoperability) are different from ours but the approach is similar: e-Vital is an open system with several interconnected modules, one of which being a multiagent system. The difference resides on the application level: when our system is a group-centred system, e-Vital is a patient-centred system (it does not use the patient's record to develop generic profiles).

### 2.4. Results of the survey

We presented three systems which are able to monitor the “elderly” in their own homes. Table 1 summarizes some features that we found relevant to compare.

All three projects seek to gather information about people by the way of hardware and software solutions. They differ in the type of collected data, in the way they use it, and in their objectives. From the simple gathering of health information for caregivers, to the complete profiling of people, resources are quite different.

In all cases, the patient is an isolated person, installed in the centre of these systems; systems which have mainly a local vision of situations.

These works have inspired more recent projects; these projects are in progress so their results can not be analysed yet. This is the case of the GERHOME [7] project, led by two French research centers. This project intends to create a smart home for weak people. This objective can also be found in the European project SOPRANO [8]. Let us also talk of the European OLDES [9] project, which tackles the problem of the elderly people access to the new technologies. It tries to create low-cost hardware with very easy-to-use interfaces.

Our research is based on the progress and technologies developed in all these projects, especially those that gather information about the monitored person, whatever granularity this information can have. For example, the information can be the cardiac output, or something of higher level like behaviour information. This data is the raw material of our system and is used to generate several categories of people. Then these categories are used to make global assumptions about people belonging to the same class.

So our problematic is to collect the results of a large number of individual monitoring and to draw several categories. This classification provides several reusable classes of people.

For that, we deploy a classification framework, usable in a large-scale configuration, and based on multiagent technology. The next section describes this architecture.

## 3. S(MA)^2^D SYSTEM

We propose a system able to carry out a generalization of profiles' patterns and to propose a classification of monitored people. S(MA)^2^D (multiagent system for keeping elderly people in their own homes) is a multiagent framework in which agents use a restricted cooperation protocol to collectively perform classifications.

### 3.1. Multiagent and health

We chose a multiagent approach because these systems proved their adequacy in many health problems [10]. In this field, medical knowledge to solve a problem can be distributed in various places. For example, to establish the medical file of a patient, it is necessary to have analyzes and tests coming from several hospitals. Agents work in various places, each agent managing a part of the knowledge.

Multiagent architecture is particularly adequate if the problem-solving implies the coordination of various specialized people (e.g., units of a hospital must collaborate to establish patient scheduling). Then, the agents have cooperative skills to communicate and to build together a solution progressively.

Moreover, many medical problems are complex and often standard solutions are not easy to find. A multiagent problem-solving is based on decomposition in subproblems. Let us take for example organ transplants [11]: when a new organ is available, the more appropriate recipient must be found very quickly. It can be located in a very far medical center. Moreover, each hospital keeps the data of its patients; they are in the waiting list depending on the type of organ. It would be difficult to conceive and apply a complex centralized system to solve this coordination problem (e.g., a standard decision aid expert system).

Multiagent technology also proved its reliability in medical information retrieval. A great quantity of medical knowledge is available on the Internet, and it is necessary to access to the most suitable information. The agents can be employed to play the mediators between doctors and patients, or between medical resources. These agents seek information issued from various sources, analyze selected data, and choose useful information according to the profiles of the consultants.

To conclude, the agents' autonomy is an adequate paradigm to deploy systems, in which each component models the behaviour of an independent entity; this entity has its own knowledge, skills, and individual goals.

We recalled the general interest of multiagent systems in the health field. Now we are going to present the expected functionalities of our system.

### 3.2. Architecture and functioning

The system is based on a bunch of sensors carried by monitored people or installed in their homes. Those sensors are, for example, presence and movement sensors or medical measuring apparatus. The data coming from sensors are transformed into indicators. Some indicators can also come from human information: notes of a nurse or patient's answers to a questionnaire.

These indicators will be used by the system to generate its classification. Their abstraction from data requires a software layer. The set of sensors and this software layer are out of the scope of our work. It is the result of projects described in Section 2.

It is important to note that the functioning of the system is independent of the type and the number of indicators.

Indicators are collected by classification agents constituting the system. Because the system is strongly distributed, indicators of two people will not be inevitably collected by the same agent. There can also be some overlaps, if the same information is collected by several agents.

**Figure 1:**
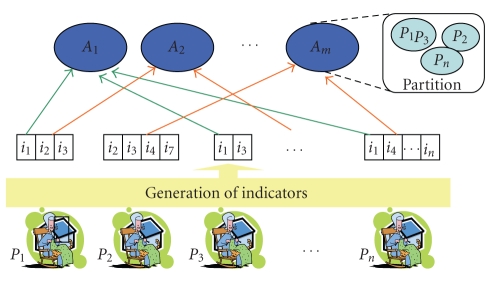
S(MA)^2^D architecture.

Thus, classification agents *A_j_* have indicators *i_k_* concerning several individuals *P*_i_ (Figure 1).

With its indicators, each agent calculates a local, partial classification. This classification does not take into account all the indicators and is related to a reduced sample of the population.

Since the data inputs are numerical values, any statistical classification method is applicable.

To refine this classification, the agents communicate each other. They congregate in acquaintances network according to the similarity of the produced partitions. More precisely, each agent seeks the other agents which made a classification close to its own. To compute the classes of the collaboratively determined partition, we designed a restricted cooperation protocol in three steps: call for participation/acquaintance's group constitution/multiagent classification.

Section 3.3 gives a detailed example of this protocol.

**Figure 2:**
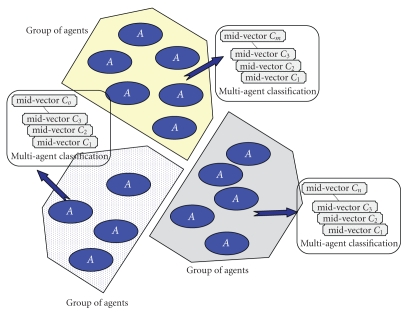
Multiagent classification.

There may be several groups of agents. They constitute parallel classifications: they are views of the same monitored people but according to various criteria (Figure 2).

### 3.3. Example

It is assumed that the behaviour indicators have numerical values. These values can be normalized by several methods as

(i) normalization between [0 ⋯ 1]

(1) $${\overset{˜}{I}}_{j}=\frac{I_{j}-I_{j}^{\text{min}}}{I_{j}^{\text{max}}-I_{j}^{\text{min}}},$$

where *I_j_* ^min^ (resp., *I_j_* ^max^) is the minimum value (resp., maximum value) of indicator number *j*, and

(ii) linear normalization

(2) $${\overset{˜}{I}}_{j}=\frac{I_{j}-{\overset{¯}{I}}_{j}}{\sigma_{j}},$$

where ${\overset{¯}{I}}_{j}$ is the average values of indicator number *j* for a given agent, and *σ_j_* is the standard deviation of the indicator number *j* for a given agent

(3) $$\sigma_{j}=\sqrt{V_{j}},\;V_{j}=\frac{1}{n}\overset{n}{\underset{k=1}{\sum }}(I_{j}^{k}-{\overset{¯}{I}}_{j}{)}^{2},$$

where *V_j_* is the variance of indicator number *j* for a given agent; *n* is the number of people monitored by an agent;

*I_j_* ^k^ is the indicator number *j* of the person number *k*.

Thereafter we apply our proposal on an example of 3 agents, 3 behaviour indicators, and 11 people. Suppose *I*_1_ is the body temperature, *I*_2_ is the number of getting up/sleeping in one night, and *I*_3_ is the number of entries to the toilets each day.

The following table shows the distribution of people (*P_i_*) and indicators (*I_j_*) on the agents (*A_k_*) of the system.

Table 2 shows that agent *A*_1_ monitors 2 indicators *I*_1_ and *I*_2_ on people *P*_1_, *P*_2_, *P*_3_, *P*_4_, *P*_5_, and *P*_11_. Agent *A*_2_ monitors 2 indicators *I*_2_ and *I*_3_ on people *P*_4_, *P*_5_, *P*_6_, *P*_7_, *P*_8_, and *P*_11_. *A*_3_ monitors 2 indicators *I*_1_ and *I*_3_ on people *P*_3_, *P*_6_, *P*_9_, *P*_10_, and *P*_11_.

This table also shows that people do not have the same indicators (it will often happen in real situations). For example, *P*_1_ has only two indicators because for this person it is not necessary to test the number of entries to the toilets. The aim is that each person has the indicators suited to his case.

We assume that the sensors send data to the system on a daily basis. In reality there are indicators that are more important than others, for example, body temperature is more important than the outside temperature, so we give a weight for each indicator; this weight will help us later to form the groups of agents and to calculate the distance between classes. The most important indicator will be the one with the largest weight.

In our case we give to *I*_1_ (body temperature) the weight 3, *I*_2_ the weight 2, and *I*_3_ the weight 1 (which is the default value).

By applying a local classification method (e.g., ISODATA [12]) each agent builds its partition. Each class is characterized by a midvector calculated by ISODATA.

**Figure 3:**
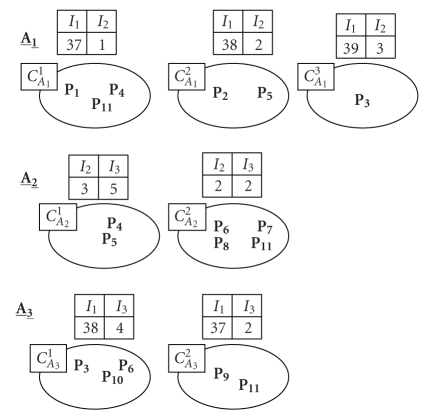
Local classification.

Preliminary step: *construction of partitions* (Figure 3).

The first step is the *call for participation*. It aims to form groups of agents to generalize the classification. The agents of the system communicate with each other through the facilitator agent. The process to constitute agents groups for each agent *A_i_* is as follows:

- *A_i_* sends its indicators to other agents;
- *A_i_* receives the indicators from other agents;
- for each other agent, *A_i_* calculates the sum of the weights of common indicators (calling *S*_1_), and the sum of the weights of noncommon indicators (calling *S*_2_);
- if *S*_1_ *≻* *S*_1_ *A_i_* responds to the agent concerned;
- the agents of a group are agents who have exchanged messages between them.

In our case, *A*_1_ sends *I*_1_ and *I*_2_. *A*_1_ also receives from *A*_2_ and *A*_3_ their indicators. We find

(4) $$\begin{array}{ll} A_{1}\cap A_{2} & =I_{2},\\ A_{1}\cap A_{3} & =I_{1},\\ A_{2}\cap A_{3} & =I_{3}. \end{array}$$

And as the weight of *I*_1_ is greater than *I*_2_ and *I*_3_, *A*_1_ chooses *A*_3_ to form a group. The result is two groups of agents. The first group is formed by *A*_1_ and *A*_3_, and the second is formed by *A*_2_.

This second step is *the acquaintance's group constitution*.

The third (and last) step is to *generalize the classification*. The agents of a group measure the distances between their classes using the weighted Euclidean distance:

(5) $$d_{w}(c,c^{\prime })={\left(\underset{1\leq j\leq n}{\sum }w_{j}\cdot {\left(d_{j}(c,c^{\prime })\right)}^{2}\right)}^{1/2}.$$

In which *c* and *c*′ are two classes, *w_j_* is the weight of the indicator number *j*, *n* is the number of common indicators between the two classes, and *d_j_*(*c*, *c*′) is the distance between the two midvectors of the two classes according to the indicator number *j*.

We can apply this formula on the actual values or normalized values of indicators. In this example we use the actual values. The agent *A*_1_ seeks to each of its classes, the closest classes of its group among other agents.

Calculation of distances between classes:

(6) $$\begin{array}{c} d_{w}(C_{A1}^{1},C_{A3}^{1})=\sqrt{3};\;\;d_{w}(C_{A1}^{1},C_{A3}^{2})=0;\\ d_{w}(C_{A1}^{2},C_{A3}^{1})=0;\;\;d_{w}(C_{A1}^{2},C_{A3}^{2})=\sqrt{3};\\ d_{w}(C_{A1}^{3},C_{A3}^{1})=\sqrt{3};\;\;d_{w}(C_{A1}^{3},C_{A3}^{2})=2\sqrt{3}. \end{array}$$

After the calculation of distances, we find that the class *C*_*A*1_^1^ should be merged with *C*_*A*3_^2^, and that *C*_*A*1_^2^ should be merged with *C*_*A*3_^1^ By contrast, *C*_*A*1_^3^ should not be merged with *C*_*A*3_^1^ because there is another class from *A*_1_ nearest to *C*_*A*3_^1^.

**Figure 4:**
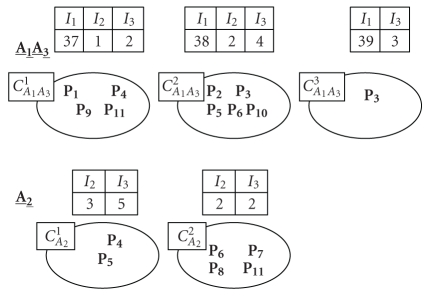
Result of the classification.

The new classes thus obtained (Figure 4) have new midvectors. These vectors are the averages of the indicators values of people belonging to the same class.

A person may belong to several classes according to the indicators used. For example, *P*_3_ is classified by *A*_1_ and *A*_3_ in a class by itself according to *I*_1_ and *I*_2_, and it is classified with *P*_2_, *P*_5_, *P*_6_, and *P*_10_, according to *I*_1_, *I*_2_, and *I*_3_.

As prospects, we intend to set a minimum threshold for the distance between classes. This threshold will be based on indicators and their weights. If the distance between two classes is greater than this threshold, they will not merge, even if they are close in the sense described above. It will be a more true-to-life approach.

### 3.4. Relevance of the multiagent architecture

This classification is actually multiagent because the classification result is not the work of a simple agent, as it is the case in other multiagent systems (choice of the most skilled [13]). It is really a collective work.

This multiagent classification answers to the problem of the search of patterns in an open and dynamic environment. Classical methods do not make it possible to increase the system scale: for example, when the number of entries changes (with the addition of a new indicator), all calculation and generation of classes must be made again.

Thus, our method satisfies the requirements of our application because it does not depend on the type of the indicators and does not require preliminary categories.

The management of the monitored people continues throughout the functioning of the system, as the agents collect more indicators values. Thus patterns evolve and the class of people can change.

Also an indicator can be deactivated: it corresponds to a data for which it is not essential to monitor this type of people.

## 4. APPLICATIONS RELATED TO HEALTH

Our system builds dynamic classifications of monitored people according to indicators that depend on the application.

This adaptability is the result of two essential characteristics. The first is the dynamic evolution of classifications—if needed, new data and new indicators can be added at any moment, and the system is able to reconfigure its classes and generate new classification patterns. The second is that the system is generic with respect to indicators and, thus, is able to function on any type of applications having strongly distributed entries.

Such a system is likely to bring solutions to several current problems in the home-monitoring field. Some of these problems are presented in this section.

**Monitoring of dependent old people**

Organizations of assistance to elderly people often use an evaluation grid of the dependence degree to determine the service needed by people. The result of this evaluation is also used to evaluate the cost of taking charge of someone.

The use of our classification system will make it possible to see whether there is an adequacy between the evaluation of monitored people by the grid and the produced profile classes. The matching of the two evaluation ways would validate our approach but also could consolidate the relevance of the grid criteria. In the contrary case, it will be necessary to re-examine the classification method and/or the selected indicators.

After validation, the system will be able to follow the evolution of the dependence degree of someone. Thus, somebody leaving his original pattern to enter a new one could be re-evaluated by the helper organization, and the assistance could be adapted to his new behaviours.

**Detection of global medical problems**

A metamonitoring will also make it possible to detect more global problems. The migration of a lot of people from a class toward another or the modification of certain characteristics of a class should indicate a collective event which affects several people; this can happen, for example, during a heat wave or an epidemic.

**Remote monitoring of people suffering from chronic health problems**

This help is for already detected people, suffering of cardiac and pulmonary insufficiencies, asthma, or Alzheimer disease.

The possibility of having a global vision of several monitored people can bring richer and more relevant information on the follow-up; the distribution in classes and the historic of the patterns evolution (system training) should allow new people entering the system to get a better service; in particular, more appropriate alerts according to the incurring risk should be generated.

**Preventive control of high-risk people**

In the long term, with the evolution of life ways, we can consider the monitoring of healthy people with personal or family antecedents relating to a disease or a medical event.

The system will make it possible to identify evolution diagrams of health parameters and life way (e.g., state-of-the-immune system, sleeping, nutrition, activity, etc.) who will indicate high risks to develop diseases.

## 5. CONCLUSION

We chose to tackle the home-monitoring issue in a more global way rather than in an only individual-centred way. This collective vision makes it possible to release individuals' patterns who will allow the system answering current health problems.

This large-scale and global solution (uninterrupted monitoring of hundreds of people) requires setting up a strongly distributed and dynamic system. Because classical classification methods are not adapted to this context, we had to propose a new distributed classification method.

The multiagent S(MA)^2^D system implements this method. To evaluate its performances, we randomly generated a great number of numerical vectors of values and we observed the formation of classes.

Now, we have to define the real indicators to take into account. One of our professional partners *CVital* (platform of coordination of care and services to the person) is making a study about people whom this organism follows. This study will make it possible to define the number and the types of main indicators.

We will also request them to semantically interpret the classes.

## Figures and Tables

**Table 1:** Overview of the three projects.

Project			
Criteria	PROSAFE	AILISA	e-Vital
Smart home equipment	Yes	Yes	No
	Equipment is installed in hospitals and residences of elderly people	Health smart homes	

Body wear equipment	Yes	Yes	No
	Accelerometer (or GPS)	Smart shirts with fall sensors	

Medical equipment	Yes	Yes	Yes
	Digital entries acquisition module	Wrist arterial pressure Pulse oximeter	Appropriate monitoring devices

Detected emergencies and supervised risks	Accidents Falls Escapes	Some medical risks Falls	Scheduled care Vital signs defection

Target	Elderly or handicapped people Patient with Alzheimer disease	Elderly people Handicapped people	People with chronic diseases

Project range (home, living environment)	At home and in hospital	At home	Living environment (that is at home but also in mobile situations)

Risk-detection method	Sensors and statistical methods	Mainly hardware	Data management and interpretation made by a multiagent system

Scale (how many people are concerned)	The system focuses on one patient but the profiling can be used in a more large scale system	The System is providing an individual help	

Links to personal medical data	Yes	No	Patient's electronic health record are stored in a hospital database (but this database is only used by the project)

Medical validation	Tested in three hospitals (three other sites are planned)	Planned in three hospitals	Tests take place in four European pilot sites

Ethical and psychological aspects	Technical mediation between health caregivers and patients	Technical mediation between caregivers and patients Psychiatric aspects	Not mentioned

Operational or experimental	Experimental	Experimental	Experimental

**Table 2:** Distribution people/indicators.

People∖Indicators	*I*_1_	*I*_2_	*I*_3_
*P*_1_	**A_1_**	**A_1_**	—
*P*_1_	**A_1_**	**A_1_**	—
*P*_3_	**A_1_**, $\underline{A_{3}}$	**A_1_**	$\underline{A_{3}}$
*P*_4_	**A_1_**	**A_1_**, *A*_2_	*A*_2_
*P*_5_	**A_1_**	**A_1_**, *A*_2_	*A*_2_
*P*_6_	$\underline{A_{3}}$	*A*_2_	*A*_2_, $\underline{A_{3}}$
*P*_7_	—	*A*_2_	*A*_2_
*P*_8_	—	*A*_2_	*A*_2_
*P*_9_	$\underline{A_{3}}$	—	$\underline{A_{3}}$
*P*_10_	$\underline{A_{3}}$	—	$\underline{A_{3}}$
*P*_11_	**A_1_**, $\underline{A_{3}}$	**A_1_**, *A*_2_	*A*_2_, $\underline{A_{3}}$

## References

[1] McMorrow K (2004). *The Economic and Financial Market Consequences of Global Ageing*.

[2] Chan M, Campo E, Estève D PROSAFE, a multisensory remote monitoring system for the elderly or the handicapped.

[3] http://www.laas.fr/laas/.

[4] Noury N (2005). Ailisa: assessment units for medical monitoring and assistance technologies in gerontology. *Gérontologie et Société*.

[5] Noury N, Villemazet C, Barralon P, Rumeau P Ambient multi-perceptive system for residential health monitoring based on electronic mailings Experimentation within the AILISA project.

[6] Sakka E, Prentza A, Lamprinos IE, Leondaridis L, Koutsouris D Integration of monitoring devices in the e-Vital service.

[7] http://gerhome.cstb.fr/.

[8] http://www.tunstall.co.uk/main.aspx?PageID=38.

[9] www.oldes.eu.

[10] Moreno A, Nealon J (2003). *Application of Software Agent Technology in the Health Care Domain*.

[11] Moreno A, Valls A, Bocio J (2001). A multi-agent system to schedule organ transplant operation. *Inteligencia Artificial: Revista Iberoamericana de Inteligencia Artificial*.

[12] Jensen JR (1996). *Introductory Digital Image Processing: A Remote Sensing Perspective*.

[13] Mukhopadhyay S, Peng S, Raje R, Palakal M, Mostafa J (2003). Multi-agent information classification using dynamic acquaintance lists. *Journal of the American Society for Information Science and Technology*.

